# Response to: ‘Medical students’ preference for returning to the clinical setting during the COVID‐19 pandemic’

**DOI:** 10.1111/medu.14283

**Published:** 2020-07-22

**Authors:** Rohin K. Reddy, Emilia G. Palmer

**Affiliations:** ^1^ Hull York Medical School University of York Heslington York UK

Editor – As fourth‐year medical students in the United Kingdom who have had to adapt to working from home amidst the nationwide cancellation of clinical placements, we read with interest Compton et al’s survey assessing the prevalence of medical students’ preference for re‐entering clinical environments during the coronavirus disease 2019 (COVID‐19) pandemic.^1^

The finding that approximately two‐thirds of surveyed students expressed a preference to return to clinical placements resonates with us.^1^ Following the cancellation of undergraduate clinical learning activities, our institution provided a series of online problem‐based learning seminars to ensure we met our learning objectives. Many other laudable innovations have since been reported.^2^ Despite novel approaches, we believe nothing can replace the integration of history taking, physical examination and clinical reasoning with decision making, empathy and professionalism in the authentic contexts that clinical placements provide.^3^ We therefore agree with the majority of surveyed students in hoping for a timely return to placements.^1^

An important finding was that students preferring to return were significantly more likely to agree with the statements, ‘It is part of my professional responsibility,' ‘I am part of the team ...' and ‘It is part of my moral obligation.'^1^ Furthermore, students in the ‘Return’ group were more likely to be autonomously motivated. This reflects Dornan et al’s ‘experience‐based learning’ model, in which, through participation, students achieve practical competence and a positive mind state that includes confidence, motivation and a sense of professional identity.^4^ Greater competence reinforces positive mind states and vice versa, resulting in a virtuous cycle whereby learning begets further learning through integration within the clinical team.

Conversely, reluctance or a perceived inability to participate can result in a vicious cycle whereby progress is impeded. At times, we have felt like spare parts swallowed up by the complex machinery of clinical environments, particularly in the early stages of our training. These feelings are surely magnified during a health care crisis and may explain the finding that students were least likely to prefer to return if they were in their first clinical clerkship year.^1^ This feeling of non‐belonging is echoed by the increased agreement in the ‘Not return’ group with the statements, ‘I don’t want to be a drain on clinicians’ time’ and ‘I am not properly trained.'^1^ The authors’^1^ logistic regression model encompassing these elements was highly predictive of preference to ‘Not return’ and highlights the need to provide extra support to these students and address the root causes of lower confidence and motivation to participate within clinical settings in order to ensure future cohorts of doctors are not disadvantaged by the COVID‐19 pandemic.
